# Rapid Development and Testing of Behavioral Text Message Reminders for Antidepressant Adherence via Online Panels: Survey Study

**DOI:** 10.2196/86605

**Published:** 2026-07-23

**Authors:** Tia R Tropea, Steven C Marcus, Amy Bucher, Cadence F Bowden, Mark Olfson, Rebecca E Stewart

**Affiliations:** 1 Department of Psychiatry, Perelman School of Medicine, University of Pennsylvania, 3535 Market Street, 3rd Fl, Philadelphia, PA, 19104, United States, 1 (215) 898-0457; 2 School of Social Policy & Practice, University of Pennsylvania, 3701 Locust Walk, Philadelphia, PA, United States; 3 Behavioral Reinforcement Learning Lab (BReLL), Lirio, Inc., Knoxville, TN, United States; 4 Department of Psychiatry, Vagelos College of Physicians and Surgeons, Columbia University, New York, NY, United States; 5 New York State Psychiatric Institute, New York, NY, United States

**Keywords:** SMS text message reminder, medication adherence, antidepressants, behavior change techniques, artificial intelligence, AI, intervention development

## Abstract

**Background:**

SMS text message reminders have been used to promote many health behaviors, such as improving diet and physical activity, managing chronic health conditions, reminding patients about medical appointments, and supporting medication adherence across a range of health conditions. Despite their promise, developing effective reminders tailored to specific patient populations is resource-intensive. AI may facilitate item development, and online research panels may provide an efficient way to test message content with target users prior to implementing large-scale trials.

**Objective:**

This study aimed to (1) develop a library of antidepressant adherence–promoting SMS text messages that are perceived as helpful, (2) test whether an online panel approach can be used to evaluate them, and (3) identify message characteristics perceived as most helpful by patients with depression taking antidepressant medication.

**Methods:**

In total, 83 SMS text message reminders were developed based on barriers to adherence and behavior change technique pairings, with approximately half authored by the study team, and half generated by AI. Using an online panel, we recruited 181 American adults with depression currently prescribed an antidepressant medication. Each participant rated a subset of messages on how much they thought each would help them remember to take their medication. Associations between message characteristics and ratings were estimated using generalized linear models in Stata. Survey weights were used in analyses to align the sample with national antidepressant user demographics.

**Results:**

The online panel was able to rapidly recruit a sample of participants, who provided 7520 item ratings in total. AI-generated messages were rated as significantly more helpful than those authored by humans (adjusted mean difference 0.24 on a 5-point scale, 95% CI 0.12‐0.36; *P*<.001). Messages addressing delayed symptom benefit were preferred over other adherence barriers, and behavior change techniques emphasizing self-monitoring (*P*<.001), habit formation (*P*<.001), and natural consequences (*P*<.001) received significantly higher ratings than those using external influence or support. No difference was observed between motivational and informational message content.

**Conclusions:**

Online panels offer a rapid, scalable approach to evaluating SMS text message reminders for patients currently taking antidepressants. When provided with specific instructions and human-led examples, AI can efficiently generate message content perceived to be helpful in promoting medication adherence. Given that AI-generated content received higher ratings than human-authored messages, future work may consider using this tool to support rapid intervention development. In addition, identifying common barriers to adherence and applying behavior change techniques to address those barriers can inform targeted message development and support adherence. Taken together, these findings demonstrate the utility of combining low-cost methods such as online panel research with AI to accelerate the design and preliminary evaluation of digital health interventions.

## Introduction

SMS text message reminders have been used to promote many health behaviors, including improving diet and physical activity, managing chronic health conditions, reminding patients of medical appointments, and medication adherence [1-4]. The scalability, low cost, and ability to reach people in real time make SMS text messages a feasible and effective strategy for health behavior change. However, crafting and validating helpful reminder messages that address the needs of specific patient populations is challenging and commonly involves recruiting and testing with target users [5]. Using traditional research methods that require patient recruitment can be time-consuming, costly, and resource-intensive.

Online panels offer a potentially rapid means of efficiently evaluating message content with target populations before committing to a full-scale evaluation of SMS text message reminder effectiveness. These large, diverse standing panels of research volunteers allow investigators to quickly recruit broadly representative samples and assess message content [6]. If successful, this approach could significantly reduce the cost and time required to gather participant feedback compared to traditional pretesting methods [7]. Although widely used in market research, online panels have been less often used in the development of digital health behavior interventions.

Evidence from systematic reviews and randomized controlled trials demonstrates the effectiveness of SMS text message reminders in promoting medication adherence across a range of health conditions [8,9]. Given this demonstrated utility, SMS text messaging may play a role in the support of antidepressant adherence. Antidepressant nonadherence is prevalent, with many patients discontinuing their medication early or taking it inconsistently, limiting its therapeutic effectiveness [10-12]. Early discontinuation is common, partly because antidepressants often require several weeks before subjective improvement occurs [13]. Tailored SMS text message reminders during this period offer an opportunity to deliver private, real-time, short-term support that might help patients maintain treatment adherence until symptom relief reinforces continued medication use.

Antidepressant nonadherence has many causes, such as forgetfulness, that may be modifiable [14,15]. Self-monitoring, social support, highlighting consequences, and fostering habit formation offer structured approaches that could address these causes [16]. SMS text messages that target these strategies of behavior change may influence message effectiveness in supporting antidepressant adherence. In addition to studying the feasibility of online panels, we were interested in comparing the perceived helpfulness of AI-generated and human-authored content. While there is some evidence suggesting that large language models such as the generative pretrained transformer series perform comparably to or better than humans on health message generation, other studies report mixed results regarding readability and empathy [17-20]. Given these inconsistent findings, it is important to directly compare AI- and human-authored message content to specifically support antidepressant adherence, which might be able to further accelerate the development and evaluation of SMS text messages.

In this study, we used an online panel to evaluate the perceived helpfulness and associated underlying behavioral mechanisms of messages designed to improve medication adherence among patients with depression receiving antidepressant treatment.

## Methods

### Overview

We conducted a cross-sectional survey in January 2025 to evaluate the perceived helpfulness of SMS text message reminders designed to promote antidepressant adherence. Messages were developed by the study team and AI and were informed by known barriers to medication adherence and evidence-based behavior change techniques (BCTs) [16]. Participants who self-reported a depression diagnosis and a current antidepressant prescription were asked to rate a subset of these messages on their perceived helpfulness. Analyses were conducted using survey-weighted regression models to examine associations between message characteristics and ratings.

### Message Development

To guide message development, we first identified known modifiable barriers to antidepressant adherence, such as forgetting doses [21]. A content expert then drew on an established framework of evidence-based BCTs to pair each barrier with an appropriate intervention strategy [16,22]; for example, forgetfulness was paired with the strategy of prompting or cueing medication-taking behavior. Each message was designed using a single barrier and BCT pairing based on the capability, opportunity, and motivation–behavior framework [23].

To facilitate comparison of AI- and human-authored content, 2 sets of SMS text message reminders were developed. First, we drafted 37 messages based on barrier and BCT pairings. Barriers were later grouped into 4 categories: social stigma, unawareness of need for continued medication adherence, delayed benefit or no immediate relief (frustration with slow effects), and forgetfulness and lack of routine. BCTs were also grouped into 4 categories: action planning and habit formation (building routines and plans), self-monitoring and feedback (tracking progress), natural consequences (highlighting outcomes of behavior), and external support and influence (engaging physicians, mental health professionals, peers, or social norms). We then provided GPT-4 (OpenAI) with these examples along with the associated barriers and BCTs and prompted it to generate 46 additional messages. Multimedia Appendix 1 provides the prompts used to generate the messages. In total, 83 messages were created, and all were reviewed for accuracy, clarity, and adherence to the specified barrier and BCT pairing.

We classified messages as informational if the content conveyed a factual statement (eg, “Did you know that one in ten adult Americans take antidepressant medications?”) and motivational if the message prioritized encouragement to support adherence (eg, “It takes a lot of strength to seek care for depression.”). Each message was then coded according to 4 characteristics: AI status (human vs AI generated), message type (motivational vs informational), barriers to adherence addressed, and BCT used. The complete set of messages, along with their barrier-BCT pairings and additional coded characteristics, can be found in Multimedia Appendix 2.

### Panel Participants

Once the full set of messages was developed, we evaluated their perceived helpfulness on antidepressant adherence using the CloudResearch Connect online platform, which provides access to a vetted panel of adult US residents. The platform enables rapid recruitment by allowing researchers to define eligibility criteria; stratify by demographic characteristics; and manage participant screening, enrollment, and compensation while maintaining participant anonymity [24]. Participants were eligible if they resided in the United States, spoke English, were aged 21 to 64 years, provided informed consent, and self-reported a diagnosis of depression and use of antidepressant medication. Within 131 minutes, the platform recruited a sample of 181 adults. Recruitment quotas were used to ensure sufficient sample sizes for subgroup analyses by sex and educational level, both of which are associated with differences in antidepressant use and adherence [25].

Eligible individuals were directed to a Qualtrics-hosted survey (Qualtrics International Inc) accessible via computer or mobile device where they completed a 10-minute questionnaire. Each participant was randomly assigned 41 or 42 of the 83 total messages and asked to rate how much each message would have helped them remember to take their antidepressant medication on a 5-point Likert scale ranging from 1 (“not at all”) to 5 (“very much”).

### Ethical Considerations

The University of Pennsylvania Institutional Review Board determined this study to be exempt from review (857419). All participants provided informed consent and received US $3 for completing the questionnaire. Participant information was anonymized on the online platform prior to data download by the research team.

### Statistical Analysis

We conducted survey-weighted analyses using Stata (StataCorp). We addressed the minimal missing data for key covariates (sex, race, ethnicity, marital status, income, and educational level) using hot-deck imputation, whereas missing age values were replaced with the sample mean. Iterative proportional fitting was applied using the *svycal* command to align the sample with national population benchmarks of adults with depression in the United States [26]. The resulting raked weights were applied in all subsequent analyses. We report weighted demographic characteristics of the survey sample, as well as comparisons to the national profile of individuals diagnosed with depression who were prescribed antidepressant medication.

To identify key factors that predicted how messages performed, we estimated unadjusted associations between message characteristics and item ratings using survey-weighted generalized linear models that account for clustering of responses within individuals. Adjusted models included AI status (human or machine generated), message type (informational or motivational), barriers (social stigma, unawareness of need for continued medication adherence, delayed benefit, and forgetfulness and lack of routine), and BCTs (self-monitoring and feedback, external influences and support, natural consequences, action planning, and habit formation) while controlling for demographic variables (age, sex, race, ethnicity, marital status, and income). Marginal means and contrasts were computed to examine differences across groups. All analyses were conducted at the item response level accounting for clustering of responses within individuals. We calculated survey-adjusted mean ratings for each message and identified the 5 highest-rated and 5 lowest-rated items.

## Results

A total of 181 participants completed the survey, yielding 7520 message ratings. After weighting, 53% of participants were aged 46 years or older, 73% were female, 92% were White individuals, 94% were non-Hispanic individuals, and nearly 59% had lower than a bachelor’s degree (Table 1).

**Table 1.** Weighted and unweighted sociodemographic characteristics of the survey sample compared to the national population of antidepressant users.

Characteristic	Unweighted survey sample (N=181), n (%)	National sample^a^^,^^b^ %)	Weighted survey sample^c^^,^^d^(%)
Age (y)			
≤45	145 (80.1)	43	47
≥46	36 (19.9)	57	53
Sex			
Male	96 (53)	28.7	26.7
Female	85 (47)	71.3	73.3
Race			
Black or African American	16 (8.8)	5.1	4.7
White	151 (83.4)	91	91.6
Other or multiple races reported	14 (7.7)	3.9	3.6
Ethnicity (self-report)			
Hispanic	14 (7.7)	6.6	6.1
Non-Hispanic	167 (92.3)	93.4	93.9
Marital status			
Married	53 (29.3)	50.5	46.9
Unmarried	128 (70.7)	49.5	53.1
Income^e^			
Poor or negative	27 (14.9)	22.3	27.8
Low income	43 (23.8)	13.5	12.6
Middle income	62 (34.3)	29.4	27.3
High income	49 (27.1)	34.8	32.4
Educational level			
Bachelor’s degree or higher	88 (48.6)	37	41.4
Lower than a bachelor’s degree	93 (51.4)	63	58.6

a All demographic categories except for educational attainment were dervived from the national estimates of antidepressant users [26].

b National education percentages reflect the 2021 U.S. Census Bureau Educational Attainment Data given educational attainment estimates were unavailable in the national antidepressant users dataset [27].

c Survey weights were constructed using raking to align the sample with national population estimates for age, sex, race, ethnicity, marital status, income, and educational level.

d Weighted percentages account for the weighting procedure and may not correspond directly to observed sample frequencies. National estimates represent population-level percentages and are provided as reference values without corresponding frequencies.

e Income categories reflect percentages of the federal poverty level based on the 2024 US Department of Health and Human Services Poverty Guidelines [28]*.*

Table 2 shows that messages generated by AI were rated as significantly more helpful than those written by humans (adjusted mean difference 0.24, 95% CI 0.12‐0.36; *P*<.001). Among barriers to adherence, messages addressing delayed benefit or no immediate effect (adjusted mean difference 0.13, 95% CI 0.004‐0.25; *P*=.04) were rated as more helpful than messages addressing unawareness of need for continued medication adherence. Our results also revealed that BCTs such as self-monitoring and feedback (adjusted mean difference 0.48, 95% CI 0.33‐0.62; *P*<.001), action planning and habit formation (adjusted mean difference 0.43, 95% CI 0.29‐0.57; *P*<.001), and natural consequences (adjusted mean difference 0.47, 95% CI 0.33‐0.61; *P*<.001) were rated as significantly more helpful than those using external support and influence.

**Table 2.** Unadjusted and adjusted mean item scores by message characteristic and demographic group (181 individuals)^a^.

	Responses (n=7520), n (%)	Adjusted mean item score (1-5; 95% CI)	*P* value	Adjusted mean difference (95% CI)	*P* value
AI status			<.001		
Human generated	3459 (46.0)	2.88 (2.69 to 3.07)		Reference	—^b^
Machine generated	4061 (54.0)	3.12 (2.97 to 3.27)		0.24 (0.12 to 0.36)	<.001
Message type			.42		
Motivational	3750 (49.9)	3.03 (2.87 to 3.19)		0.05 (–0.07 to 0.16)	.42
Informational	3770 (50.1)	2.99 (2.81 to 3.16)		Reference	—
Barriers to adherence			.13		
Social stigma	888 (11.8)	2.97 (2.77 to 3.17)		0.02 (–0.15 to 0.19)	.80
Unawareness of need for continued medication adherence	1762 (23.4)	2.95 (2.77 to 3.13)		Reference	—
Delayed benefit or no immediate relief	2565 (34.1)	3.07 (2.93 to 3.22)		0.13 (0.004 to 0.25)	.04
Forgetfulness and lack of routine	2305 (30.7)	3.01 (2.80 to 3.21)		0.06 (–0.03 to 0.06)	.18
Behavior change technique			.001		
Self-monitoring and feedback	1957 (26.0)	3.10 (2.90 to 3.28)		0.48 (0.33 to 0.62)	<.001
External support and influence	1250 (16.6)	2.63 (2.46 to 2.80)		Reference	—
Natural consequences	2288 (30.4)	3.10 (2.94 to 3.26)		0.47 (0.33 to 0.61)	<.001
Action planning and habit formation	2025 (26.9)	3.06 (2.86 to 3.26)		0.43 (0.29 to 0.57)	<.001

a Analyses were conducted at the person-item level. Unadjusted models used survey-weighted generalized linear models accounting for clustering of items within individuals. Adjusted models additionally controlled for the message and demographic characteristics shown in the table.

b Not applicable.

Table 3 highlights the 5 highest-rated and 5 lowest-rated messages. The most highly rated items were predominantly motivational messages focused on overcoming forgetfulness. In contrast, the lowest-rated items were all human authored and inspirational in tone and emphasized external support or influence from health care providers.

**Table 3.** Participant ratings of SMS text message reminders for antidepressant adherence.

Message	Source	Message type	Barrier to adherence	Behavior change technique	Score (1-5), mean (SD)
Highest-ranked messages					
It takes a lot of strength to seek care for depression. Feel proud of what you’re doing for your health! Take your meds today.	Human	Motivational	Social stigma	Self-monitoring and feedback	3.66 (1.18)
You’ve taken so many positive steps toward managing your health! Keep it up. Take your meds today.	Human	Motivational	Forgetfulness and lack of routine	Self-monitoring and feedback	3.60 (1.15)
You’re making progress every day you take your meds. Keep up the good work and take them today.	Machine	Motivational	Delayed symptom relief and lack of immediate benefit	Self-monitoring and feedback	3.59 (1.07)
Antidepressants can help restore interest in activities you once enjoyed. Support your progress and take your meds today.	Machine	Informational	Forgetfulness and lack of routine	Self-monitoring and feedback	3.52 (1.02)
Your dedication to treatment makes a real difference. Support yourself and take your meds today.	Machine	Motivational	Forgetfulness and lack of routine	Self-monitoring and feedback	3.43 (1.08)
Lowest-ranked messages					
Did you know that one in ten adult Americans take antidepressant medications? Join them in taking care of your mental health. Take your meds today.	Human	Informational	Delayed symptom relief and lack of immediate benefit	External support and influence	2.54 (1.36)
Pharmacists can suggest ways to improve your experience with medication. If you need help, talk to them. Take your meds today.	Human	Informational	Delayed symptom relief and lack of immediate benefit	External support and influence	2.46 (1.25)
Medication questions? Get answers! Ask your provider via the Penn portal, call their office, or talk to a pharmacist. Take your meds today.	Human	Informational	Delayed symptom relief and lack of immediate benefit	External support and influence	2.43 (1.35)
Have questions about your medications? Your providers can help. Send a message or call them to discuss any concerns. Take your meds today.	Human	Informational	Delayed symptom relief and lack of immediate benefit	External support and influence	2.39 (1.17)
Worried people may judge you for taking antidepressants? It’s more likely they’ll admire you for taking care of your health! Take your meds today.	Human	Motivational	Social stigma	External support and influence	2.18 (1.36)

## Discussion

This study demonstrates the feasibility of using online research panels to rapidly evaluate SMS text message reminders for antidepressant adherence. In just a few hours of fielding the survey, participants provided item-level helpfulness ratings across a large library of candidate reminder messages. Helpfulness ratings were generally high, although they varied by message source, barrier focus, and BCT. These results suggest that using online panels and AI provides a fast and low-cost [29] method for pretesting intervention messaging content.

A notable finding was that, when guided by examples developed by content experts within a structured framework of barriers and BCTs, AI was able to produce highly rated and sometimes preferred reminder messages targeting antidepressant adherence. This pattern aligns with emerging evidence that AI can support or improve health message design when prompts incorporate theoretical and domain-specific guidance [18]. Although further investigation is needed, one possible explanation for the observed preference for AI-generated messages may be large language models’ tendencies to produce responses that are agreeable or aligned with expectations [30]. As a result, AI models may be perceived as more relevant or appealing to users. To determine whether this preference translates into actual adherence, a randomized controlled trial is currently underway [31]. While future research is needed to confirm these findings, the results of this study suggest a scalable path to expanding message libraries while maintaining acceptability, which could support the design of personalized health messaging interventions.

Our analysis revealed important message preferences with implications for digital interventions and clinical practice. Messages that emphasized overcoming forgetfulness, developing a medication-taking routine, and highlighting downstream consequences were generally rated as more helpful than those emphasizing reliance on external support and influence. These results support prior work indicating that forgetfulness and disrupted routines are leading modifiable causes of unintentional nonadherence, with behavioral models underscoring habit formation and self-regulation as drivers of consistent medication use [12,32,33].

Messages encouraging patients to contact their health care providers were consistently among the lowest rated. While further study is needed, this finding is consistent with prior research showing that patients often view physicians and mental health professionals' communication as burdensome, time-consuming, or unhelpful in the context of medication adherence support [34]. Our findings may also reflect the tendency for individuals to attribute their health behavior to self-management efforts while underestimating environmental or interpersonal factors (eg, physician and mental health professional support) in shaping health behavior [35]. Importantly, this does not suggest that clinician communication is ineffective as previous work has demonstrated the positive effect that physician and mental health professional advice can have on health behavior change [36]. Rather, this tendency may help explain the observed preference for self-directed over physician- and mental health professional–mediated strategies and inform clinical guidelines on effective strategies for adherence counseling. For example, adherence interventions might be more successful if they focus on empowering patients with self-monitoring tools and highlight the benefits of consistent medication use. In general, autonomy-supportive and habit-based strategies have been demonstrated to improve adherence by strengthening patients’ self-efficacy and integrating medication taking into daily routines [33]. Applying these strategies alongside clinician support may provide the strongest benefit for health behavior change and represents an important area for further research.

Several limitations should be acknowledged. First, participants recruited through online panels may not be fully representative of all patients initiating antidepressant treatment. Additionally, the study team was unable to validate participants’ treatment histories and relied on self-report to determine study eligibility; therefore, it is possible that some participants may have misreported their diagnosis or medication use. Second, our evaluation focused on perceived helpfulness rather than actual behavioral outcomes, which may limit interpretability given that individuals’ intentions are imperfect predictors for their actual health behavior [37]. Although the response patterns appear credible, we have not yet tested whether any of these messages improve medication adherence. A randomized controlled trial is currently underway to address this limitation [31,38]. Third, although message development was grounded in prior theoretical considerations and empirical research, we may have missed some factors that meaningfully influence antidepressant adherence. Furthermore, the distinction between motivational and informational messages was based on criteria established by the study team; future research should explore more empirically grounded approaches to improve the reliability of this classification. Finally, the generalizability of these findings to other populations, health behaviors, and conditions remains unknown. While the methods appear broadly applicable for rapid message testing across various health interventions, the specific content preferences observed for antidepressant adherence may not generalize to other conditions or health behaviors. Furthermore, given that the sample was limited to adults aged 21 to 64 years as part of the eligibility criteria for a subsequent randomized controlled trial, the findings may not reflect the reminder needs and preferences of older adults.

Despite these limitations, the results suggest that online panels can offer a scalable, efficient approach for rapidly evaluating health behavior change messages. In addition, guided AI can play a productive role in message development. Together, these methods offer a practical approach for accelerating the design and pilot-testing of digital health interventions and reducing associated costs [39]. As SMS text messaging and related digital strategies continue to expand across health domains, the ability to efficiently identify and refine effective messages through these methods could accelerate advances in the field.

## Supplementary material

10.2196/86605 Multimedia Appendix 1 AI prompting procedure for message generation.

10.2196/86605 Multimedia Appendix 2 Complete set of messages along with their barrier–behavior change technique pairings and coded characteristics.

## Abbreviations

BCT: behavior change technique
