# Supplementary material for: Rapid Development and Testing of Behavioral Text Message Reminders for Antidepressant Adherence via Online Panels: Survey Study
Source: J Med Internet Res. 2026 Jul 23;28:e86605. doi: 10.2196/86605 (PMC13395428; doi:10.2196/86605)
Supplement: Multimedia Appendix 1 [file jmir-v28-e86605-s001.docx]

Multimedia Appendix 1. AI Prompting Procedure for Message Generation

The AI-generated messages were developed using ChatGPT-4 through an investigator-guided prompting process. The prompts below are representative of those used during the message development process in the ChatGPT interface. The study team provided ChatGPT with a spreadsheet of investigator-developed example messages, each labeled by adherence barrier and behavior change technique.

Step 1. Initial context prompt

“We are developing text message reminders for adults with depression who are taking antidepressant medication. The goal is to help patients remember to take their medication. The messages should be brief, appropriate for SMS delivery, supportive, nonjudgmental, and focused on medication adherence.

Step 2. Framework prompt

“The messages are organized by barriers to antidepressant adherence and behavior change techniques. Barriers include social stigma, lack of awareness of the need for continued medication, delayed benefit or lack of immediate relief, and forgetfulness/lack of routine. Behavior change techniques include action planning/habit formation, self-monitoring/feedback, natural consequences, and external support/influence.”

Step 3. Message-generation prompt

“Please use the attached examples as models. Generate 50 additional text message reminders similar in tone, length, and structure. For each new message, provide the message text, the adherence barrier it addresses, and the behavior change technique it uses.”

Step 4. Refinement prompt

“Please revise the messages you just generated to ensure they are concise, easy to understand, and appropriate for people taking antidepressants. Avoid overly clinical language, avoid sounding judgmental, and avoid messages that imply the patient has failed if they miss a dose.”

Step 5. Review and selection

The study team reviewed the generated messages for accuracy, clarity, redundancy, tone, and consistency with the assigned barrier and behavior change technique. Messages that were repetitive, unclear, inconsistent with the intended construct, or otherwise unsuitable were discarded or edited by the investigators before inclusion in the final message library.
